# Improved Treatment of Hydrocele

**Published:** 1843-06

**Authors:** 


*Improved Treatment of Hydrocele.*—It need scarcely be recalled to mind that in the operation for hydrocele, after the serum has been discharged through the canula of the trocar, it is usual to inject an irritating fluid in order to induce an adhesive inflammation in the parietes of the tunica vaginalis. The inflammation somewhat subsides after about the fifth day; but a month commonly elapses before the whole quantity of the injected fluid is absorbed and a cure effected. On this somewhat tedious course of practice Lisfranc has made the following improvement:—On the sixth day after the use of a vinous injection, he makes a second puncture, for the purpose of emptying the tunica vaginalis of all the accumulated liquid, thus sparing nature the task of its absorption; and by these means he alleges that a cure can be completed in less than half the time occupied by the usual method.—*Lond. Lancet*, from *Bulletin de Thérapeutique.*

